# Elastofibroma dorsi: determinants of symptom burden, bilaterality, and perioperative morbidity in 102 surgically treated patients

**DOI:** 10.1186/s12893-026-03770-x

**Published:** 2026-04-27

**Authors:** Caner İşevi, Burçin Çelik, Mehmet Gökhan Pirzirenli, Faruk Ömür, Hilkat Fatih Elverdi, Yasemin Büyükkarabacak

**Affiliations:** Faculty of Medicine, Department of Thoracic Surgery, Ondokuz Mayis University, Samsun, Turkey

**Keywords:** Elastofibroma dorsi, Scapula, Chest wall, Soft tissue neoplasms, Occupational exposure, Postoperative complications, Risk factors

## Abstract

**Objectives:**

Elastofibroma dorsi (ED) is a rare, benign, tumor-like soft-tissue lesion typically located in the subscapular region. Despite having a characteristic radiological appearance, clinical presentation and early postoperative course may vary from patient to patient. This study aimed to describe the clinical and pathological characteristics of surgically treated ED and to explore factors associated with symptom burden and early postoperative course (seroma and length of stay).

**Materials and methods:**

We conducted a retrospective analysis of 102 patients who underwent surgical resection for histopathologically confirmed ED between January 2010 and June 2025. Data on demographics, symptom profiles, occupational history, imaging and pathological measurements, early postoperative course (seroma and length of hospital stay) were collected and analyzed.

**Results:**

Women constituted 85% of patients (*n* = 87), and the average age was 61.5 ± 7.9 years. Seventy-five (73.5%) were symptomatic, and 51 (50%) had bilateral ED. The most common occupation was homemaker (62.7%), and the most prevalent comorbidity was hypertension (36.3%). Postoperative seroma occurred in 19 (18.6%) patients. Symptomatic patients were significantly younger (*p* < 0.001), more likely to have bilateral lesions (*p* < 0.001), and had larger pathological tumor volumes (*p* = 0.023) and areas (*p* = 0.002). In multivariable analysis, female sex (OR 0.136 for male sex; 95% CI, 0.033–0.564; *p* = 0.008) and younger age (OR 0.933 per year; 95% CI, 0.881–0.988; *p* = 0.017) were independently associated with bilateral disease. Lesions were predominantly located ipsilateral to the dominant hand (*p* < 0.001). Seroma development was exclusive to symptomatic patients (*p* = 0.009) and was associated with longer symptom duration (*p* = 0.027). In contrast, length of hospital stay correlated with preoperative tumor volume (rho = 0.411; *p* < 0.001) and area (rho = 0.401; *p* < 0.001).

**Conclusion:**

Symptom burden in elastofibroma dorsi was associated with disease burden, particularly bilaterality and larger pathological tumor dimensions. Postoperative seroma was associated with clinical factors (symptomatic presentation and longer symptom duration), whereas length of stay correlated mainly with tumor size. These findings may support individualized perioperative counseling and risk stratification in surgically treated patients.

**Trial registration:**

ClinicalTrials.gov, NCT07010224. Registered on 30 May 2025. Retrospectively registered.

**Supplementary Information:**

The online version contains supplementary material available at 10.1186/s12893-026-03770-x.

## Introduction

Elastofibroma dorsi (ED) is a slow-growing, benign, non-encapsulated soft tissue lesion typically located in the subscapular space between the inferior pole of the scapula and the posterior thoracic wall. First described by Jarvi and Saxén in 1961 [1, 2]. The high prevalence of pre-elastofibroma changes in autopsy studies and the incidental detection of ED on thoracic computed tomography (CT) in up to 2% of the elderly population suggest that ED may be an underdiagnosed, age-related degenerative process [3, 4].

While pain and a subscapular mass are common findings, the reported incidence of asymptomatic cases varies widely, from 1% to 16% [4–6]. The clinical presentation of ED is variable. Many patients are asymptomatic, while others report a palpable mass, pain during shoulder movements, or a snapping sensation of the scapula. The mean age at diagnosis ranges from 49 to 66 years, with a marked female predominance [2, 7, 8]. Although repetitive mechanical friction between the scapula and the thoracic wall is the most widely accepted theory, the precise etiology of ED remains not fully understood [5].

ED is often unilateral but can be bilateral in 10% to 66% of cases [6, 9, 10]. Surgical excision is generally indicated for symptomatic lesions or those ≥ 5 cm in diameter that cause functional or cosmetic concerns [11]. The most frequently reported postoperative complications are hematoma and seroma, with rates as high as 40% to 50%, particularly after resection of large tumors [5, 12, 13].

Despite increasing recognition of ED, most published data derive from case reports or small case series. Consequently, clinicopathologic factors associated with symptom development and early postoperative morbidity remain incompletely characterized.

The purpose of this study was to analyze demographic, clinical, and pathological characteristics in a large, surgically confirmed cohort of patients with ED and to identify factors associated with symptom burden and early postoperative morbidity.

We hypothesized that (1) greater disease burden, reflected by bilaterality and larger tumor dimensions, would be associated with higher symptom burden at presentation, and (2) lesion laterality would correlate with hand dominance, supporting a potential mechanical contribution to disease distribution.

## Material & method

### Study design and ethical approval

This single-center retrospective cohort study was conducted at a tertiary thoracic surgery department and included patients who underwent surgical resection for elastofibroma dorsi. The study protocol was approved by the Institutional Review Board (Approval No: 2025/231, Approval Date: April 17, 2025). All procedures adhered to the principles of the Declaration of Helsinki. Written informed consent was obtained from all patients for both the surgical treatment and the use of their clinical data for research and publication purposes.

The study was registered on ClinicalTrials.gov (Registration No: NCT07010224, Registered on 30 May 2025).

Given the extended 15-year study period, potential variations in imaging protocols, surgical technique, and perioperative management over time were considered inherent limitations of the retrospective design.

### Patient selection

A total of 102 consecutive patients who underwent surgery for ED between January 2010 and June 2025 were included in the study. The inclusion criteria were as follows:

- Age ≥ 18 years,
- Surgical resection for ED performed at our institution,
- Histopathological confirmation of the ED diagnosis,
- Minimum of one month of postoperative follow-up,
- Complete availability of preoperative imaging, operative notes, and pathology reports.

The exclusion criteria were:

- A diagnosis based solely on clinical or radiological findings without surgical confirmation,
- Inconclusive histopathology or an alternative final diagnosis,
- Missing essential (imaging, surgical, or follow-up) data,
- Pediatric patients (< 18 years),
- Lack of documented informed consent.

No randomization was performed due to the retrospective design of the study. No patients were lost to observation.

During the study period, additional patients with imaging findings consistent with ED were managed conservatively and were not included in the present analysis. The current study intentionally focused on surgically treated patients in order to ensure histopathological confirmation and allow evaluation of early postoperative morbidity.

### Indications for surgery

Surgical excision was generally recommended for patients with:

- Persistent or progressive symptoms (pain, snapping, swelling, movement limitation)
- Functional impairment
- Cosmetic concern
- Large lesions (commonly ≥ 5 cm)
- Diagnostic uncertainty on imaging

A subset of patients (26.5%) were asymptomatic at presentation. In these cases, surgery was performed primarily due to large lesion size, patient preference, cosmetic considerations, or diagnostic uncertainty.

In ten patients, elastofibroma dorsi was identified incidentally during thoracotomy performed for lung cancer. In these cases, the lesion was encountered intraoperatively and excised during the same surgical session based on preoperative patient preference and intraoperative assessment. The decision to remove the lesion was not driven by suspicion of metastatic disease but rather by patient request and the opportunity for concurrent excision.

### Data collection

Data were systematically extracted from the hospital’s Health Information Management System, surgical records, Picture Archiving and Communication System (PACS), and pathology reports using a standardized data collection form adapted from previous studies on ED [2, 5, 9].

Demographic characteristics, presenting symptoms, occupational history, comorbidities, imaging findings, surgical details, pathological measurements, and early postoperative outcomes were recorded.

Follow-up information was obtained from outpatient clinic documentation and available imaging records. Owing to the retrospective design, postoperative follow-up was not standardized according to a predefined protocol, and long-term surveillance was not systematically performed. Consequently, recurrence analysis was limited to documented clinical encounters during routine care and was not considered a primary endpoint of the study.

To ensure data accuracy, two investigators independently extracted the data, and any discrepancies were resolved by consensus.

### Variables and groupings

Data on the following variables were collected to evaluate clinical features, etiologic associations, and surgical outcomes in patients with ED:

#### Demographic data

Age and sex were recorded for all patients.

#### Clinical characteristics

- Data on symptomatology, including the presence of pain, swelling, a snapping sensation, and movement limitation, were recorded.
- The snapping phenomenon was defined as an audible or palpable friction between the scapula and thoracic wall during active shoulder abduction, confirmed by physical examination.
- Symptom duration was documented for each patient.
- Lesion laterality (right, left, or bilateral) were recorded.
- Hand dominance was determined based on patient self-report or clinical history noted in medical records.
- Smoking history was recorded.

To provide a quantitative assessment of overall clinical symptomatology, a composite variable termed Symptom Count was defined for each patient. The presence of four cardinal symptoms—pain, swelling, snapping, and limitation of shoulder movement—was evaluated based on clinical examination and patient history. Each symptom present was assigned one point, yielding a total score ranging from 0 (asymptomatic) to 4 (all symptoms present).

Symptom Count was analyzed both as a continuous variable and categorically in relation to demographic and pathological parameters.

All primary analyses were performed at the patient level. In cases of bilateral disease, tumor size–related variables were aggregated to reflect total tumor burden per patient.

#### Occupational and lifestyle factors

Occupational history was recorded and categorized into predefined professional groups. Engagement in physically demanding activities, including agricultural labor, was documented to explore potential associations with lesion distribution and morphology.

#### Imaging modalities

- Preoperative imaging modalities included ultrasonography, computed tomography (CT), and/or magnetic resonance imaging (MRI).
- Tumor dimensions were measured on CT and/or MRI using the hospital’s PACS software.
- All linear and volumetric measurements were based on the findings reported in the original CT and/or MRI radiology reports.

**Fig. 1:**
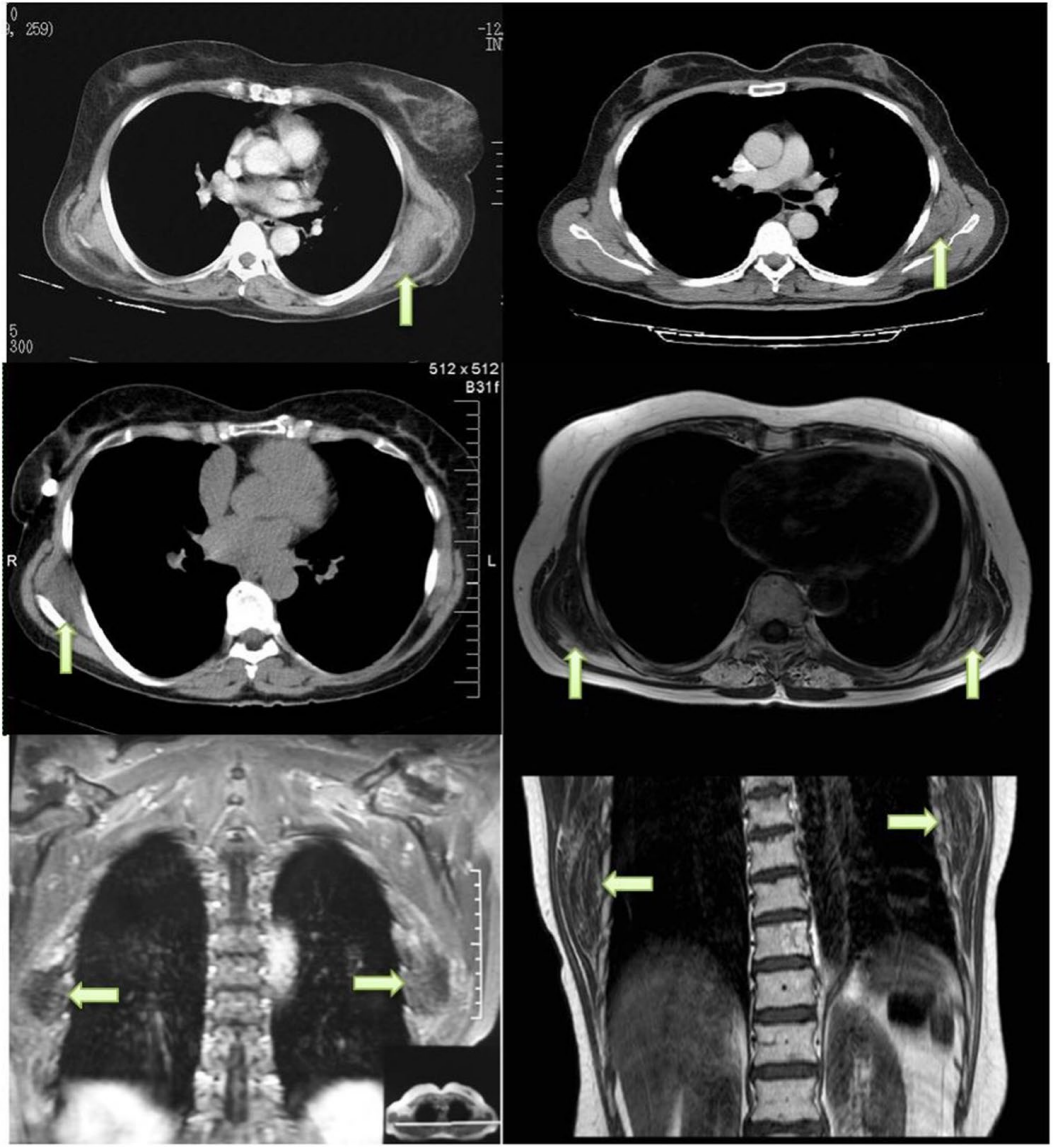
Representative computed tomography (CT) and magnetic resonance imaging (MRI) scans of elastofibroma dorsi. Axial CT images (upper and middle rows, left panels) show poorly encapsulated, soft-tissue masses located between the lower scapula and posterior chest wall (arrows). The lesions demonstrate heterogeneous attenuation with interspersed fat density strands. Corresponding axial, coronal, and sagittal T1- and T2-weighted MR images (right panels) reveal a well-defined, heterogeneous mass with alternating fibrous (low signal) and fatty (high signal) areas, characteristic of elastofibroma dorsi

Representative preoperative CT and MRI images of elastofibroma dorsi are shown in Fig. 1. The lesions typically appeared as poorly encapsulated, heterogeneous soft-tissue masses located between the inferior scapular border and the posterior chest wall, exhibiting alternating fibrous and fatty attenuation on CT and corresponding low- and high-signal areas on MRI.

Axial CT images (upper and middle rows, left panels) show poorly encapsulated, soft-tissue masses located between the lower scapula and posterior chest wall (arrows). The lesions demonstrate heterogeneous attenuation with interspersed fat density strands. Corresponding axial, coronal, and sagittal T1- and T2-weighted MR images (right panels) reveal a well-defined, heterogeneous mass with alternating fibrous (low signal) and fatty (high signal) areas, characteristic of elastofibroma dorsi.

#### Surgical and histopathologic data

- Final tumor dimensions and volumes were obtained from postoperative histopathology reports.
- Histopathologic examination was performed in all cases to confirm the diagnosis of ED.
- Tumor surface area (cm²) was calculated as the product of the longest and shortest axes. Tumor volume (cm³) was calculated as the product of the three orthogonal dimensions (length × width × depth).

The primary unit of analysis was the patient. For patients with bilateral elastofibroma dorsi, size-related variables (area and volume) were summarized at the patient level as total tumor burden, calculated by summing measurements from both sides. Clinical outcomes (symptom burden, seroma, length of stay) were analyzed per patient.

**Fig. 2:**
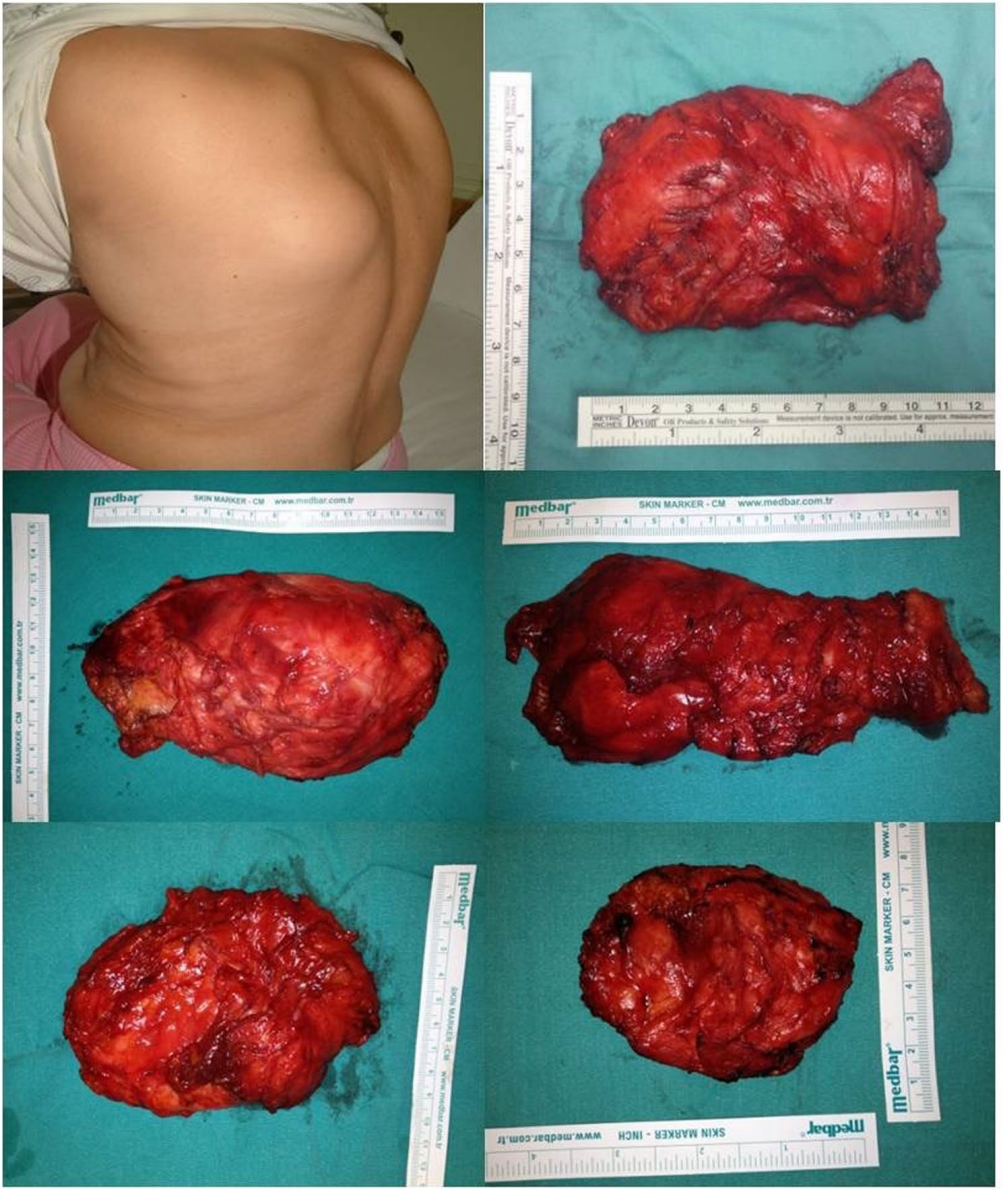
Clinical appearance and gross specimen of elastofibroma dorsi. Preoperative image shows a subscapular bulging mass on the posterior thoracic wall. The resected specimens demonstrate poorly encapsulated, rubbery, and lobulated soft-tissue masses with alternating fibrous and fatty areas, consistent with the characteristic macroscopic appearance of elastofibroma dorsi

Representative postoperative clinical and gross images are presented in Fig. 2.

#### Postoperative outcomes

Early postoperative complications, including seroma, hematoma, pneumothorax, and wound infection, were assessed. Complications were further classified based on the need for subsequent intervention (e.g., aspiration).

The primary postoperative endpoints of this study were early morbidity measures, specifically seroma formation and length of hospital stay.

Because of the retrospective design and the absence of a standardized follow-up protocol, long-term outcomes—including recurrence and postoperative symptom resolution—were not systematically evaluated. Any available follow-up documentation was limited to routine clinical visits and was not used for formal recurrence analysis.

### Occupational groups and agricultural work

Patients were classified into five primary occupational groups:

1. Homemakers,
2. White-collar workers,
3. Blue-collar workers,
4. Farmers,
5. Self-employed.

Additionally, patients were stratified into two groups based on agricultural work: those who regularly participated in agricultural activities (including farmers and some homemakers) were classified as “agricultural workers.”

### Surgical technique

All surgical procedures were performed under general anesthesia using a single-lumen endotracheal tube. Patients were positioned in the lateral decubitus position. Perioperative pain management was managed with either an intercostal nerve block or a patient-controlled analgesia pump. For bilateral cases, surgeries were performed in two separate sessions with a mean interval of three weeks.

In patients with bilateral elastofibroma dorsi, a staged surgical approach was routinely preferred in our institution. This strategy was adopted to reduce postoperative morbidity, facilitate functional recovery, and minimize complications such as seroma formation and shoulder dysfunction. The timing of the second procedure was individualized based on the patient’s recovery from the first operation, but it was generally performed within 2–4 weeks. When lesions were asymmetric, the larger or more symptomatic lesion was resected first. In cases with similar lesion characteristics, the dominant side or the side associated with greater functional limitation was prioritized.

Following a skin incision over the subscapular region, the latissimus dorsi and serratus anterior muscle fibers were separated in a muscle-sparing approach to access the lesion. Marginal excision was the standard technique, however, if adherence to surrounding tissues raised suspicion for incomplete resection, the procedure was converted to a wide excision to ensure clear macroscopic margins. A closed-suction drain was routinely placed in all patients. Drain output was recorded daily, and removal was generally performed when drainage decreased to less than 30–50 mL per 24 h, typically between postoperative days 2 and 4, depending on clinical assessment. In cases with persistent output, the drain was maintained until satisfactory reduction was achieved. A layered closure of the muscle, subcutaneous tissue, and skin was performed to restore anatomical integrity.

### Statistical analysis

All statistical analyses were performed using R software (version 4.5.1). The normality of continuous variables was assessed with the Shapiro–Wilk test. Continuous variables were presented as mean ± standard deviation or median (interquartile range), as appropriate. For two-group comparisons, the Independent Samples t-test was used for normally distributed data and the Mann-Whitney U test for non-normally distributed data. For comparisons among more than two groups, one-way ANOVA and the Kruskal-Wallis H test were applied for parametric and non-parametric data, respectively. Spearman’s rank correlation was used to evaluate the association between continuous/ordinal outcomes (e.g., symptom burden, length of stay) and other continuous variables. To identify independent risk factors while controlling for confounders, a multivariable logistic regression model was constructed.

Specifically, bilateral disease was modeled as the dependent variable. Age, sex, and smoking status were included a priori as clinically relevant covariates. Adjusted odds ratios (ORs) with 95% confidence intervals (CIs) were reported.

All analyses were performed at the patient level.

A p-value < 0.05 was considered statistically significant.

To avoid sole reliance on p-values, quantitative effect estimates (mean differences, correlation coefficients, and adjusted ORs with 95% CIs) were reported to better convey the magnitude of associations.

### STROBE compliance

This manuscript was prepared in accordance with the Strengthening the Reporting of Observational Studies in Epidemiology (STROBE) statement for observational studies. This study was reported in accordance with the STROBE guidelines during manuscript drafting and revision to ensure transparent and complete reporting. The completed checklist is provided as Supplementary Material.

### Use of artificial intelligence

ChatGPT (OpenAI, San Francisco, CA, USA) was used for language editing and improvement of manuscript clarity. All outputs were reviewed, revised, and approved by the authors. The authors take full responsibility for the accuracy and integrity of the content.

## Results

Of the 102 patients included in the cohort, the mean age was 61.5 ± 7.9 years, and 87 (85.3%) were female. The general demographic, clinical, and pathological characteristics of the entire cohort are summarized in Table 1. Ten patients underwent ED excision during evaluation for lung cancer and reported no ED-related symptoms; these lesions were considered incidentally detected.

**Table 1:** Baseline demographic, clinical, and pathological characteristics of the study cohort (*N* = 102)

Characteristic	Category	Total (*N* = 102)
Continuous variables	(Mean ± SD)	
Age (years)		61.5 ± 7.9
Symptom Duration (months)		20.6 ± 33.1
Length of Hospital Stay (days)		5.4 ± 3.5
Pathological Tumor Area (cm²)		77.7 ± 41.4
Pathological Tumor Volume (cm³)		254.9 ± 185.6
Categorical Variables	n (%)	
Sex	Male	15 (14.7%)
	Female	87 (85.3%)
Bilaterality	Yes	51 (50.0%)
Morbidity (Hypertension)	Yes	37 (36.3%)
Agricultural Worker	Yes	56 (54.9%)
Smoking	Yes	27 (26.5%)
Diabetes mellitus	Yes	16 (15.7%)
Lung Cancer	Yes	10 (9.8%)
Diaphragmatic Eventration	Yes	3 (2.9%)
Preoperative Pain	Yes	58 (56.9%)
Preoperative Swelling	Yes	61 (59.8%)
Preoperative Snapping	Yes	21 (20.6%)
Preoperative Movement Limitation	Yes	10 (9.8%)
Seroma	Yes	19 (18.6%)
Need for Seroma Aspiration	Yes	14 (13.7%)
Occupation	White-collar worker	14 (13.7%)
	Farmer	6 (5.9%)
	Homemaker	64 (62.7%)
	Blue-collar worker	13 (12.7%)
	Self-employed	5 (4.9%)

Values are presented as mean ± standard deviation or n (%)

### Comparison of symptomatic and asymptomatic patients

Of the 102 patients in the cohort, 75 (73.5%) were symptomatic and 27 (26.5%) were asymptomatic. The clinical and pathological characteristics of these two groups are compared in Table 2.

**Table 2:** Comparison of clinical and pathological characteristics between symptomatic and asymptomatic patients

Characteristic	Category	Symptomatic (*n* = 75)	Asymptomatic (*n* = 27)	Test Statistic	*p*-value
Continuous variables	(Mean ± SD)				
Age (years)		59.7 ± 7.3	66.9 ± 7.4	t(40.7) = 4.22	**< 0.001**
Symptom Duration (months)		28.0 ± 35.9	0.1 ± 0.4	W = 9.5	**< 0.001**
Length of Hospital Stay (days)		5.0 ± 2.9	6.6 ± 4.7	W = 1233	0.092
Pathological Tumor Area (cm²)		85.0 ± 42.2	57.5 ± 32.1	W = 596.5	**0.002**
Pathological Tumor Volume (cm³)		278.8 ± 193.6	188.2 ± 144.7	W = 711.5	**0.023**
Categorical Variables	n (%)				
Sex	Male	8 (10.7%)	7 (25.9%)	-	NS
	Female	67 (89.3%)	20 (74.1%)	-	NS
Bilaterality	Yes	49 (65.3%)	2 (7.4%)	χ²(1) = 24.38	**< 0.001**
Morbidty (Hypertension)	Yes	34 (45.3%)	3 (11.1%)	χ²(1) = 8.63	**0.003**
Agricultural Worker	Yes	39 (52.0%)	17 (63.0%)	-	NS
Smoking	Yes	18 (24.0%)	9 (33.3%)	-	NS
Diabetes mellitus	Yes	14 (18.7%)	2 (7.4%)	-	NS
Lung Cancer	Yes	0 (0.0%)	10 (37.0%)	(Fisher’s)	**< 0.001**
Preoperative Pain	Yes	57 (76.0%)	1 (3.7%)	χ²(1) = 39.41	**< 0.001**
Preoperative Swelling	Yes	60 (80.0%)	1 (3.7%)	χ²(1) = 44.95	**< 0.001**
Preoperative Snapping	Yes	21 (28.0%)	0 (0.0%)	χ²(1) = 7.88	**0.005**
Preoperative Movement Limitation	Yes	10 (13.3%)	0 (0.0%)	(Fisher’s)	0.06
Seroma	Yes	19 (25.3%)	0 (0.0%)	(Fisher’s)	**0.003**
Need for Seroma Aspiration	Yes	14 (18.7%)	0 (0.0%)	(Fisher’s)	**0.02**
Occupation	White-collar worker	13 (17.3%)	1 (3.7%)	-	NS
	Farmer	3 (4.0%)	3 (11.1%)	-	NS
	Homemaker	45 (60.0%)	19 (70.4%)	-	NS
	Blue-collar worker	10 (13.3%)	3 (11.1%)	-	NS
	Self-employed	4 (5.3%)	1 (3.7%)	-	NS

Values are presented as mean ± standard deviation or n (%). Statistical comparisons were performed using the independent samples t-test (t), Mann-Whitney U test (W), Chi-square test (χ²), or Fisher’s exact test. *NS* Not significant

Asymptomatic patients who underwent surgery had either large lesions, diagnostic uncertainty, or concomitant oncologic evaluation requiring histopathological confirmation, rather than symptom-driven indications.

### Comparison of unilateral and bilateral cases

The cohort was evenly divided between patients with unilateral (*n* = 51, 50.0%) and bilateral (*n* = 51, 50.0%) disease. A comparison of the demographic, clinical, and pathological variables between these groups is summarized in Table 3.

**Table 3:** Comparison of demographic, clinical, and pathological variables between unilateral and bilateral cases

Characteristic	Category	Total (*n* = 102)	Unilateral (*n* = 51)	Bilateral (*n* = 51)	Test Static	*p*-value
Continuous variables	(Mean ± SD)					
Age (years)		61.5 ± 7.9	63.5 ± 8.4	59.7 ± 7.0	W = 1600.5	**0.008**
Symptom Duration (months)		20.6 ± 33.1	13.8 ± 27.2	27.4 ± 37.2	W = 705.5	**< 0.001**
Pathological Tumor Area (cm²)		77.7 ± 41.4	58.3 ± 28.6	97.1 ± 43.4	W = 989.5	**0.037**
Pathological Tumor Volume (cm³)		254.9 ± 185.6	213.7 ± 177.5	296.1 ± 186.0	W = 610	**< 0.001**
Categorical Variables	n (%)					
Symptomatic status	Yes	75 (73.5%)	26 (51.0%)	49 (96.1%)	χ²(1) = 24.38	**< 0.001**
Sex	Male	15 (14.7%)	12 (23.5%)	3 (5.9%)	-	NS
	Female	87 (85.3%)	39 (76.5%)	48 (94.1%)	χ²(1) = 5.00	**0.025**
Hypertension	Yes	37 (36.3%)	13 (25.5%)	24 (47.1%)	χ²(1) = 4.24	**0.04**
Lung cancer	Yes	10 (9.8%)	10 (19.6%)	0 (0.0%)	χ²(1) = 8.98	**0.003**
Preoperative pain	Yes	58 (56.9%)	17 (33.3%)	41 (80.4%)	χ²(1) = 21.14	**< 0.001**
Preoperative swelling	Yes	61 (59.8%)	25 (49.0%)	36 (70.6%)	χ²(1) = 4.08	**0.043**
Tumor location	Right	59 (57.8%)	35 (68.6%)	24 (47.1%)	χ²(1) = 4.02	**0.045**
	Left	43 (42.2%)	16 (31.4%)	27 (52.9%)	-	NS

Values are presented as mean ± standard deviation or n (%). Statistical comparisons were performed using the Mann-Whitney U test (W) or the Chi-square test (χ²). *NS* Not significant

A strong ipsilateral association was identified between the dominant hand and the side of the lesion. Among left-hand–dominant patients, 94.1% had a lesion on the left side, whereas 68.2% of right-hand–dominant patients had a lesion on the right side (χ² (1) = 20.10, *p* < 0.001). No significant differences were observed between the dominant hand groups in continuous variables, including age, tumor size, or length of hospital stay (all *p* > 0.05). However, asthma was observed exclusively in the left-hand–dominant group (17.6% vs. 0%; Fisher’s *p* = 0.004).

### Independent predictors of bilateral disease

A multivariable logistic regression model was constructed to identify independent predictors of bilateral involvement, including age, sex, and smoking status as covariates. The model revealed that female sex was a strong independent predictor of bilateral disease, with male patients being significantly less likely to present with bilateral lesions (OR = 0.136; 95% CI, 0.033–0.564; *p* = 0.008). Additionally, younger age was associated with an increased probability of bilaterality; each one-year increase in age was associated with a 6.7% decrease in the odds of bilateral involvement (OR = 0.933; 95% CI, 0.881–0.988; *p* = 0.017). Smoking status did not significantly influence the risk of bilateral disease (*p* = 0.355).

### Surgical and histopathological findings

Representative imaging examples, and gross specimen of elastofibroma dorsi are shown in Figs. 1 and 2.

### Concordance analysis of preoperative and pathological measurements

**Fig. 3:**
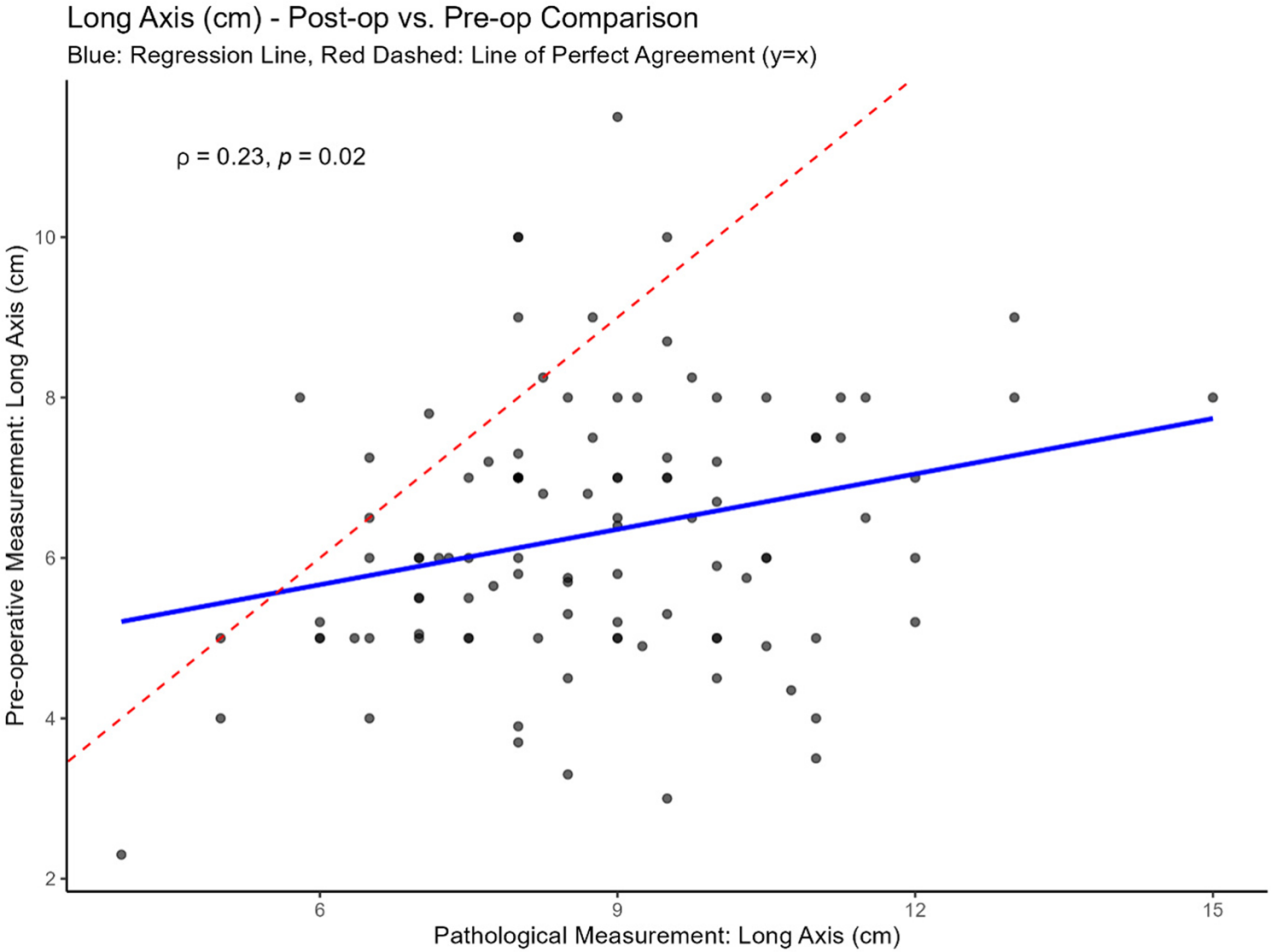
Correlation between preoperative and pathological long axes (ρ = 0.23, *P* = 0.02); solid line indicates linear fit; shaded area, 95% CI. Abbreviations: CI, confidence interval

A modest but statistically significant positive correlation was observed between the preoperative long axis and the pathological long axis (ρ = 0.23, *p* = 0.02) (Fig. 3). While the correlation coefficient is modest (rho = 0.23), it demonstrates a consistent trend of radiological underestimation, which is crucial for surgical planning.

### Clinical and demographic characteristics by occupational groups

The study population was classified into five occupational groups, the largest of which was housewives (*n* = 64, 62.7%). The remaining groups were white-collar workers (*n* = 14, 13.7%), blue-collar workers (*n* = 13, 12.7%), farmers (*n* = 6, 5.9%), and self-employed individuals (*n* = 5, 4.9%). The descriptive characteristics for each group are presented in Table 4.

**Table 4:** Baseline characteristics of the study population according to occupational groups

Characteristic	Category	Total (*N* = 102)	White-collar worker (*n* = 14)	Farmer (*n* = 6)	Homemaker (*n* = 64)	Blue-collar worker (*n* = 13)	Self-employed (*n* = 5)	*p*-value
Continuous variables	(Mean ± SD)							
Age (years)		61.5 ± 7.9	59.8 ± 7.8	57.5 ± 10.4	62.2 ± 7.6	63.2 ± 8.3	59.0 ± 7.7	0.451^a^
Pathological Tumor Volume (cm³)		254.9 ± 185.6	270.0 ± 189.5	209.7 ± 123.8	251.9 ± 190.7	290.0 ± 210.7	212.9 ± 134.8	0.945^b^
Categorical Variables	n (%)							
Sex	Male	15 (14.7%)	2 (14.3%)	3 (50.0%)	0 (0.0%)	9 (69.2%)	1 (20.0%)	**< 0.001** ^**c**^
	Female	87 (85.3%)	12 (85.7%)	3 (50.0%)	64 (100.0%)	4 (30.8%)	4 (80.0%)	
Symptomatic status	Yes	75 (73.5%)	13 (92.9%)	3 (50.0%)	45 (70.3%)	10 (76.9%)	4 (80.0%)	0.392^c^
Bilaterality	Yes	51 (50.0%)	10 (71.4%)	2 (33.3%)	30 (46.9%)	6 (46.2%)	3 (60.0%)	0.637^c^
Smoking	Yes	27 (26.5%)	1 (7.1%)	3 (50.0%)	12 (18.8%)	7 (53.8%)	4 (80.0%)	**< 0.001** ^**c**^
Agricultural worker	Yes	56 (54.9%)	3 (21.4%)	5 (83.3%)	43 (67.2%)	5 (38.5%)	0 (0.0%)	**< 0.001** ^**c**^
Preoperative snapping	Yes	21 (20.6%)	8 (57.1%)	1 (16.7%)	10 (15.6%)	2 (15.4%)	0 (0.0%)	**0.013** ^**c**^
Raynaud’s phenomenon	Yes	1 (1.0%)	0 (0.0%)	0 (0.0%)	0 (0.0%)	0 (0.0%)	1 (20.0%)	0.062^c^

Data are presented as mean ± standard deviation (SD) or n (%)

^a^ One-way ANOVA (F (4,94) = 0.93); ^b^ Kruskal–Wallis χ²(4) = 0.75; ^c^ Fisher’s exact test

### Symptom profile and burden

#### Pain symptom

The presence of pain was most strongly associated with bilaterality; 70.7% of patients with pain had bilateral disease, compared with 22.7% of those without (*p* < 0.001). Pathological tumor dimensions were also significantly larger in patients with pain, including tumor area (*p* = 0.008) and volume (*p* = 0.028). Pain was strongly associated with other preoperative symptoms, including swelling (*p* = 0.001) and movement limitation (*p* = 0.004).

An interesting finding was the significant association between pain and left-sided tumors (*p* = 0.041).

#### Swelling symptom

Preoperative swelling, observed in 59.8% of patients (*n* = 61), was associated with younger age (59.7 ± 7.0 vs. 64.3 ± 8.5 years; *p* = 0.001) and longer symptom duration (29.6 ± 38.3 vs. 7.3 ± 16.1 months; *p* < 0.001).

Pathologically, tumor length, area, and volume were all significantly larger in patients with swelling (all *p* < 0.05). Bilaterality was also more frequent in this group (59.0% vs. 36.6%; *p* = 0.043). Swelling was strongly associated with concurrent pain (*p* = 0.001) and snapping sensation (*p* = 0.014).

#### Snapping sensation

A preoperative snapping sensation was present in 20.6% of patients (*n* = 21). Its presence was significantly associated with younger age (57.6 ± 8.5 vs. 62.6 ± 7.5 years; *p* = 0.021) and longer symptom duration (42.0 ± 46.4 vs. 15.1 ± 26.4 months; *p* < 0.001). All patients with snapping (100%) were classified as symptomatic, a significantly higher proportion than in those without snapping (66.7%; *p* = 0.001).

Clinically, snapping was strongly associated with swelling (85.7% vs. 53.1%; *p* = 0.014).

The most striking finding was its disproportionately high frequency among white-collar workers, who accounted for 38.1% of the snapping group versus only 7.4% of the non-snapping group (*p* = 0.013). In contrast to pain and swelling, pathological tumor size was not significantly associated with snapping (all *p* > 0.05).

#### Movement limitation

Movement limitation, the least common symptom (9.8%), was exclusively associated with pain; all patients (100%) in this group also reported pain (*p* = 0.004). Although bilaterality was more frequent in those with limitation (80.0% vs. 46.7%), the difference did not reach statistical significance (*p* = 0.096), likely due to the small sample size (*n* = 10). No significant differences were found between groups for any continuous variables, including tumor volume (all *p* > 0.05).

#### Symptom burden (Total number of symptoms)

To provide a quantitative measure of the overall clinical burden, a composite “Symptom Count” score was created for each patient by summing the presence of the four cardinal symptoms (pain, swelling, snapping, and movement limitation). The score ranged from 0 (asymptomatic) to 4.

The distribution of symptom burden across the cohort was as follows: 26 patients (25.5%) had a score of 0, 20 (19.6%) had a score of 1, 39 (38.2%) had a score of 2, 16 (15.7%) had a score of 3, and one patient (1.0%) presented with all four symptoms.

Spearman’s correlation analysis revealed that a higher symptom count was significantly and positively correlated with longer symptom duration (ρ = 0.639, *p* < 0.001) and larger pathological tumor area (ρ = 0.316, *p* = 0.001) and volume (ρ = 0.269, *p* = 0.006). Conversely, symptom burden was negatively correlated with age (ρ = -0.353, *p* < 0.001), indicating that younger patients presented with more symptoms.

Group comparisons further showed that the mean symptom count was significantly higher in patients with bilateral disease compared to those with unilateral disease (1.9 ± 0.8 vs. 1.0 ± 1.1; *p* < 0.001). No significant differences in symptom burden were found based on sex, agricultural worker status, or the development of postoperative seroma. These findings are summarized in Table 5.

**Table 5:** Factors associated with symptom burden (Number of symptoms)

Analysis type	Variable	Value / Correlation coefficient (ρ)	*p*-value
Continuous variables	(Spearman correlation)		
	Symptom duration (months)	0.639	**< 0.001**
	Age	-0.353	**< 0.001**
	Total number of imaging studies	0.36	**< 0.001**
	Pathological tumor area (cm²)	0.316	**0.001**
	Volume discrepancy (cm³)	0.275	**0.005**
	Pathological tumor volume (cm³)	0.269	**0.006**
	Pathological tumor length (cm)	0.211	**0.034**
Categorical variables	(Mann–Whitney U test)	Mean number of symptoms (Mean ± SD)	
Bilaterality	Unilateral (No)	1.0 ± 1.1	**< 0.001**
	Bilateral (Yes)	1.9 ± 0.8	
Sex	Female (0)	1.5 ± 1.0	0.152
	Male (1)	1.1 ± 1.3	
Agricultural worker	No (0)	1.5 ± 1.0	0.53
	Yes (1)	1.4 ± 1.1	
Seroma	No (0)	1.4 ± 1.1	**0.101**
	Yes (1)	1.8 ± 0.6	

Values are presented as correlation coefficients (ρ) for continuous variables and mean ± standard deviation (SD) for categorical variables. Associations were tested using Spearman correlation or Mann–Whitney U test, as appropriate

### Postoperative outcomes

#### Postoperative seroma

The overall incidence of postoperative seroma was 18.6% (*n* = 19). Factors associated with seroma development were analyzed, and the comparative findings are summarized in Table 6.

**Table 6:** Comparison of clinical and demographic characteristics according to postoperative seroma development

Characteristic	Category	Total (*N* = 102)	No Seroma (*n* = 83)	Seroma (*n* = 19)	Test statistic	*p*-value
Continuous variables	(Mean ± SD)					
Age (years)		61.5 ± 7.9	61.9 ± 8.2	59.9 ± 6.2	-	NS
Symptom duration (months)		20.6 ± 33.1	17.7 ± 30.0	33.5 ± 42.7	W = 533.5	**0.027**
Pathological tumor volume (cm³)		254.9 ± 185.6	248.2 ± 188.7	283.9 ± 173.1	-	NS
Categorical variables	n (%)					
Symptomatic status	Yes	75 (73.5%)	56 (67.5%)	19 (100.0%)	χ²(1) = 6.82	**0.009**
Family history	Yes	10 (9.8%)	5 (6.0%)	5 (26.3%)	(Fisher’s)	**0.018**
Depression	Yes	4 (3.9%)	1 (1.2%)	3 (15.8%)	(Fisher’s)	**0.02**
Aspiration required	Yes	14 (13.7%)	0 (0.0%)	14 (73.7%)	(Fisher’s)	**< 0.001**
Bilaterality	Yes	51 (50.0%)	39 (47.0%)	12 (63.2%)	-	NS
Hypertension	Yes	37 (36.3%)	27 (32.5%)	10 (52.6%)	-	NS

Values are presented as mean ± standard deviation (SD) or n (%). Statistical comparisons were performed using the Mann-Whitney U test (W), Chi-square test (χ²), or Fisher’s exact test. *NS* Not significant

#### Length of hospital stay

The mean length of hospital stay (LOS) for the entire cohort was 5.4 ± 3.5 days. Factors associated with LOS were investigated, and the findings are summarized in Table 7. A strong positive correlation was observed between LOS and several measures of tumor size, most notably the total number of imaging studies (ρ = 0.444, *p* < 0.001), preoperative tumor volume (ρ = 0.411, *p* < 0.001), and preoperative tumor area (ρ = 0.401, *p* < 0.001). In contrast, the only categorical variable significantly associated with LOS was preoperative pain, where patients with pain had a paradoxically shorter stay (4.8 ± 2.8 vs. 6.3 ± 4.2 days; *p* = 0.049).

**Table 7:** Factors associated with length of hospital stay

Analysis type	Variable	Value / Correlation coefficient (ρ)	*p*-value
Continuous variables	(Spearman correlation)		
	Preoperative tumor volume (cm³)	0.411	**< 0.001**
	Preoperative tumor area (cm²)	0.401	**< 0.001**
	Pathological tumor area (cm²)	0.213	**0.031**
	Pathological tumor volume (cm³)	0.206	**0.038**
	Preoperative tumor depth (cm)	0.198	**0.046**
Categorical variables	(Mann–Whitney U test)	Mean number of symptoms (Mean ± SD)	
Preoperative pain	No	6.3 ± 4.2	**0.049**
	Yes	4.8 ± 2.8	
Symptomatic status	Asymptomatic	6.6 ± 4.7	0.092
	Symptomatic	5.0 ± 2.9	
Seroma	No	5.6 ± 3.7	0.546
	Yes	4.7 ± 2.4	

Values for categorical variables are presented as mean ± standard deviation (SD). Correlations were assessed using Spearman’s rank correlation; group comparisons were performed using the Mann-Whitney U test

## Discussion

In this retrospective cohort of 102 patients, three main themes emerged regarding the clinical presentation, demographic patterns, and surgical outcomes of elastofibroma dorsi: (i) symptom development was associated with younger age, bilaterality, and larger pathological tumor dimensions, reflecting a greater “disease burden”; (ii) our findings suggest the presence of different etiological influences, with occupational exposure supporting a mechanical/environmental contribution and occasional familial observations raising the possibility of structural predisposition; and (iii) postoperative outcomes appeared to diverge mechanistically, with seroma risk associated with clinical factors and length of hospital stay primarily related to the physical dimensions of the lesion.

A notable proportion of patients in our cohort were asymptomatic at presentation. In these cases, surgical excision was performed due to large lesion size, patient preference, or intraoperative detection during thoracic procedures, rather than symptom-driven indications. Therefore, our findings reflect the clinical characteristics of surgically managed ED rather than exclusively symptomatic disease.

Importantly, this study was not designed to quantify the clinical benefit of surgery in terms of symptom resolution or functional improvement. Because standardized postoperative symptom scores and validated patient-reported outcome measures were not routinely recorded in our retrospective cohort, we focused on objective early postoperative endpoints (seroma and length of stay) and on clinicopathological correlates of symptom burden at presentation. Therefore, our findings should be interpreted as hypothesis-generating regarding risk stratification and perioperative counseling rather than as evidence of long-term therapeutic effectiveness.

Our cohort’s, female predominance and older age distribution are consistent with previous series [2, 7, 8]. The clustering of asymptomatic cases in older patients aligns with the slow-growing nature of ED and the increased likelihood of incidental detection on imaging performed for other reasons [3, 4]. The high proportion of bilateral cases (50%), nearly all of whom were symptomatic, corroborates the wide range of bilateralism reported (10–66%) and supports the concept that increased “lesion burden” impairs scapulothoracic mechanics, predisposing patients to symptoms [6, 9, 10]. Consistent with this, larger pathological dimensions were also associated with symptom presence, highlighting disease burden as a strong determinant of clinical presentation [2, 7, 9, 10].

The optimal surgical strategy for bilateral elastofibroma dorsi remains a subject of debate. Previous reports have suggested staged resections, often prioritizing the larger or more symptomatic lesion, with an interval of approximately 30 days between procedures [14]. In our practice, we also favor a staged approach, as it allows better postoperative recovery, reduces the risk of complications, and facilitates functional assessment after the first operation. Simultaneous bilateral resection may theoretically reduce overall treatment time but could increase postoperative discomfort and complication risk, particularly in elderly patients. Therefore, individualized decision-making based on patient characteristics and symptom burden appears to be the most appropriate strategy. However, current evidence is largely based on small case series and expert opinion, and no standardized guidelines exist regarding the optimal surgical approach in bilateral cases.

This pattern suggests that the association between age and symptom presence may be partially mediated through bilaterality: younger patients appear more susceptible to bilateral disease, which in turn increases the lesion burden and facilitates symptom development. This hypothesis warrants testing in future studies using formal mediation analysis. The observation of right-sided predominance in unilateral cases and a shift towards left-sided predominance in bilateral cases raises the possibility of differences in ergonomics and scapulothoracic symmetry, which should be verified with objective measures of hand dominance and workload.

Symptoms, particularly pain and swelling, were positively associated with pathological tumor size. The discrepancy between imaging- and pathology-based volumes also correlated with symptom burden, suggesting that the true biological burden of ED is better captured by pathological measurements. In our study, patients with preoperative pain also had a significantly higher rate of hypertension. While this does not imply a direct pathogenetic relationship, it may indicate that comorbidity burden contributes to greater symptom severity. The literature provides no clear evidence that hypertension is an independent risk factor for ED; however, some series have reported that comorbidity burden may increase postoperative complication rates [15].

The imaging findings in our study are consistent with the literature, which describes ED as a heterogeneous, layered mass. Magnetic Resonance Imaging (MRI) is considered the most reliable modality for diagnosis [16]. The more frequent use of MRI in our patients who presented with functional symptoms such as snapping and swelling further supports its diagnostic superiority in symptomatic cases. Recent studies have demonstrated that typical imaging patterns correlate strongly with histopathological architecture, often obviating the need for preoperative biopsy [17, 18].

Occupational and familial factors appear to play complementary roles in the development of ED. In our series, significantly larger linear tumor dimensions in agricultural workers support the hypothesis that repetitive microtrauma contributes to lesion morphology. However, the lack of difference in total tumor volume suggests that growth may occur in a flatter or irregular pattern rather than through simple volumetric expansion. Interestingly, a positive family history was more frequently observed in the non-agricultural group, consistent with reports highlighting a potential hereditary pathway independent of mechanical stress [19].

Moreover, our cohort strongly confirmed the link between lesion laterality and hand dominance [20]. The finding that 94.1% of left-hand–dominant patients had a lesion on the left side, whereas 68.2% of right-hand–dominant patients had one on the right (*p* < 0.001), suggests that repetitive microtrauma from dominant-side loading may trigger ED development in a subset of patients.

These findings may suggest the presence of different etiological influences. While occupational exposure supports a mechanical hypothesis, occasional reports of familial clustering in the literature raise the possibility of structural or genetic susceptibility. However, our data are insufficient to confirm a true genetic pathway, and this interpretation should be considered hypothesis-generating. The observation that agricultural workers had altered tumor morphology (larger linear dimensions but unchanged volume) has clinical implications for surgical planning, as an irregular, lamellar growth pattern may complicate the management of dead space.

Our finding that the snapping symptom was independent of tumor size is consistent with previous reports [21, 22]. Its higher frequency among white-collar workers may reflect the influence of other biomechanical factors, such as postural stress or scapular dyskinesia, which should be clarified through advanced functional analyses.

Our findings on seroma are consistent with the lower-to-mid range of rates reported in the literature (10–43%) [5, 10, 15]. Crucially, seroma development was not associated with tumor size, but it was significantly related to the preoperative symptomatic status and longer symptom duration. The relatively moderate seroma rate observed in our cohort may partly reflect the routine use of closed-suction drainage with standardized removal criteria. These findings suggest that seroma risk cannot be explained solely by the postoperative “dead space,” but that biological background factors such as chronic inflammation and tissue reactivity may also play a determining role. The higher frequency of family history observed in some subgroups should be interpreted cautiously. While this finding may raise the possibility of underlying structural susceptibility, no genetic or molecular analyses were performed in this study. Therefore, this observation should be considered hypothesis-generating and requires validation in future investigations incorporating genetic methodologies.

In our series, the mean LOS was 5.4 ± 3.5 days, which is longer than the 1–3 days typically reported in the literature [23, 24]. This difference likely reflects institutional and contextual factors rather than increased surgical morbidity. As a tertiary referral center, a subset of our patients were concurrently evaluated for thoracic malignancies, necessitating extended perioperative monitoring. Furthermore, a substantial proportion of patients resided in geographically remote areas, where early discharge was not feasible due to logistical challenges in outpatient follow-up. Importantly, prolonged hospitalization in our cohort was primarily driven by these clinical and logistical considerations rather than severe postoperative complications.

Complete resection of elastofibroma dorsi can be challenging due to its non-encapsulated nature. In this context, surgical success relies primarily on intraoperative macroscopic assessment rather than formal margin evaluation. As highlighted in previous reports, the concept of “complete resection” in elastofibroma dorsi is largely dependent on the surgeon’s experience and judgment, since pathological assessment alone may not fully reflect completeness of excision. Regarding follow-up, there is currently no standardized protocol. However, clinical reassessment within 6–12 months has been suggested as a reasonable approach in the absence of symptoms or recurrence [14]. These considerations further emphasize the importance of individualized surgical decision-making and highlight the need for future prospective studies to establish standardized management strategies.

Overall, these findings highlight the central importance of the symptom–bilaterality–pathological size triad in guiding patient counseling and surgical planning. When estimating seroma risk, clinicians should consider symptom duration, active symptomatology, and potential systemic predisposition (e.g., family history). In contrast, expectations regarding hospital stay should be managed based on preoperative tumor size. The strengths of our study include its large single-center cohort, detailed symptom burden analysis, and reliance on pathology-based measurements.

As a surgically treated cohort, this study is inherently subject to selection bias and does not fully represent the broader spectrum of radiologically diagnosed, conservatively managed elastofibroma dorsi. The retrospective design limits causal inference and introduces potential record-based bias. Additionally, the single-center setting and the regional predominance of homemakers and agricultural workers may restrict generalizability.

Several clinically relevant variables were not systematically recorded, including body mass index, detailed workload quantification, and objective biomechanical assessments such as scapulothoracic kinematics or postural analysis. Consequently, interpretations regarding mechanical influences and symptoms such as snapping remain indirect. Imaging–pathology comparisons were based on conventional linear measurements rather than three-dimensional segmentation, and minor variability related to tissue fixation or specimen handling cannot be excluded.

The absence of a non-surgical control group precludes comparison between operative management and the natural course of conservatively managed cases. Furthermore, standardized patient-reported outcome measures, validated pain scales (e.g., VAS/NRS), and functional scores were not routinely collected, limiting evaluation of symptom severity and postoperative functional improvement. Potential confounders such as medication use (e.g., antiplatelet or anticoagulant therapy) and metabolic control variables were also not modeled.

Despite these limitations, this study contributes substantially to the literature owing to its large sample size and detailed clinico-pathological analyses. Priorities for future research should include prospective, multicenter designs that systematically evaluate mechanical factors (BMI, hand dominance, posture) and incorporate functional investigations, such as scapulothoracic kinematic analyses, to better elucidate the pathogenesis of symptoms like snapping.

## Conclusion

This large single-center cohort of surgically treated elastofibroma dorsi demonstrates that symptom burden is primarily associated with disease burden, particularly bilaterality and larger pathological tumor dimensions. Female sex and younger age were independently associated with bilateral involvement, suggesting that mechanical factors alone may not fully explain disease distribution.

Postoperative seroma formation was associated with preoperative symptomatic status and longer symptom duration, whereas length of hospital stay correlated mainly with tumor size. These findings provide practical information for perioperative risk estimation and patient counseling in surgically treated patients.

Although these results do not alter current treatment indications, they contribute to a more individualized and evidence-informed approach to the management of elastofibroma dorsi. 

## Supplementary Information

Supplementary Material 1.

## Data Availability

The datasets generated and/or analyzed during the current study are available from the corresponding author on reasonable request.

## Abbreviations

ED: Elastofibroma dorsi
CT: Computed tomography
MRI: Magnetic resonance imaging
LOS: Length of hospital stay
CI: Confidence interval
OR: Odds ratio
