# Supplementary material for: Elastofibroma dorsi: determinants of symptom burden, bilaterality, and perioperative morbidity in 102 surgically treated patients
Source: BMC Surg. 2026 Apr 27;26:405. doi: 10.1186/s12893-026-03770-x (PMC13261838; doi:10.1186/s12893-026-03770-x)
Supplement: Supplementary file 1 — Supplementary Material 1. [file 12893_2026_3770_MOESM1_ESM.docx]

The STROBE reporting checklist

For checking that observational epidemiology research articles can be understood and used by everyone

| 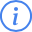 Note |
| --- |
| If you have not used a reporting guideline before, read about [how and why to use them](https:/resources.equator-network.org/about/reporting-guidelines.html) and check whether STROBE is the [most applicable reporting guideline](https:/resources.equator-network.org/reporting-guidelines/strobe/index.html?#applicability) for your work.  Reporting guidelines are most useful when used early in research. When writing a manuscript or application, consider using the [Full Guidance](https:/resources.equator-network.org/reporting-guidelines/strobe/index.html) where you’ll see explanations and examples for each item.  After writing, demonstrate adherence by completing this checklist:   1. Specify where each item is described (see [Note 1](#sec-specify)). 2. Cite this checklist (See [Note 2](#sec-cite)). 3. Include your completed checklist as a supplement when submitting to a journal so that future readers can use it to find information. |

|  | Item Description | Location (or reason for not reporting) |
| --- | --- | --- |
| **Title and abstract** |  |  |
| [1a. Indicate the study’s design](https:/resources.equator-network.org/reporting-guidelines/strobe/items/title-abstract-indicate-study-design.html?utm_source=strobe&utm_medium=checklist&utm_campaign=1_1) | Indicate the study’s design with a commonly used term in the title or the abstract. | **Location:**   - **Abstract – Methods (first sentence)** - **Materials & Methods – “Study Design and Ethical Approval”, first sentence**   **Statement:** The study design is clearly stated in the Abstract (Methods section) and reiterated in the Materials & Methods section. |
| [1b. Abstract](https:/resources.equator-network.org/reporting-guidelines/strobe/items/abstract.html?utm_source=strobe&utm_medium=checklist&utm_campaign=1_1) | Provide in the abstract an informative and balanced summary of what was done and what was found. | **Location:**  - **Abstract – Background** - **Abstract – Methods** - **Abstract – Results** - **Abstract – Conclusions**  **Statement:** The abstract provides a structured and balanced summary of the study objectives, design, population, main analyses, key findings (including effect estimates and statistical significance), and principal conclusions without overstating the results. Both positive and non-significant findings are reported to ensure transparency. |
| **Introduction** |  |  |
| [2. Background / rationale](https:/resources.equator-network.org/reporting-guidelines/strobe/items/background-rationale.html?utm_source=strobe&utm_medium=checklist&utm_campaign=1_1) | Explain the scientific background and rationale for the investigation being reported. | **Location:**  - **Background – Paragraphs 1–4** - **Background – Final paragraph (study purpose and hypotheses)**  **Statement:** The Background section provides a clear scientific context for elastofibroma dorsi, including its epidemiology, clinical presentation, proposed pathogenesis (mechanical friction and possible degenerative process), and reported postoperative complications. The limitations of existing literature—primarily small case series and lack of detailed clinicopathological correlation—are explicitly stated. The rationale for the current study is clearly justified by highlighting the need for a larger surgically confirmed cohort to evaluate determinants of symptom burden, bilaterality, and early postoperative morbidity. The study objectives and hypotheses are explicitly presented at the end of the Background section. |
| [3. Objectives](https:/resources.equator-network.org/reporting-guidelines/strobe/items/objectives.html?utm_source=strobe&utm_medium=checklist&utm_campaign=1_1) | State specific objectives, including any prespecified hypotheses. | **Location:**  - **Background – Final paragraph (Purpose statement)** - **Background – Final paragraph (Hypotheses statement)**  **Statement:** The specific objectives of the study are clearly stated in the final paragraph of the Background section. The study aims to (1) analyze demographic, clinical, and pathological characteristics in a surgically confirmed cohort of elastofibroma dorsi patients, and (2) identify factors associated with symptom burden and early postoperative morbidity.  Additionally, two prespecified hypotheses are explicitly presented:   - (1) Greater disease burden, reflected by bilaterality and larger tumor dimensions, would be associated with higher symptom burden at presentation. - (2) Lesion laterality would correlate with hand dominance, supporting a potential mechanical contribution to disease distribution. |
| **Methods** |  |  |
| [4. Study design](https:/resources.equator-network.org/reporting-guidelines/strobe/items/study-design.html?utm_source=strobe&utm_medium=checklist&utm_campaign=1_1) | Present key elements of study design early in the paper. | **Location:**  - **Abstract – Methods (first sentence)** - **Methods – “Study Design and Ethical Approval” (first paragraph)**  **Statement:** The key elements of the study design are presented early in both the Abstract and the Methods section. The study is clearly described as a single-center retrospective cohort study conducted at a tertiary thoracic surgery department, including surgically treated and histopathologically confirmed cases of elastofibroma dorsi over a defined 15-year period (January 2010–June 2025). The patient-level unit of analysis, inclusion/exclusion criteria, and primary postoperative endpoints (seroma and length of hospital stay) are explicitly defined in the Methods section. |
| [5. Setting](https:/resources.equator-network.org/reporting-guidelines/strobe/items/setting.html?utm_source=strobe&utm_medium=checklist&utm_campaign=1_1) | Describe the setting, locations, and relevant dates, including periods of recruitment, exposure, follow-up, and data collection. | **Location:**  - **Methods – “Study Design and Ethical Approval” (first paragraph)** - **Methods – “Patient Selection” (first paragraph)** - **Methods – “Data Collection” section** - **Results – first paragraph (cohort description)**  **Statement:** The study setting is clearly described in the Methods section. This was a single-center study conducted at the Department of Thoracic Surgery, Ondokuz Mayıs University Faculty of Medicine, a tertiary referral center in Samsun, Turkey.  The recruitment period spanned from January 2010 to June 2025 and included consecutive adult patients who underwent surgical resection for elastofibroma dorsi with histopathological confirmation.  Data were collected retrospectively from the hospital’s Health Information Management System (HIMS), surgical records, PACS imaging archive, and pathology reports using a standardized data extraction form.  Postoperative follow-up was based on routine outpatient visits and available clinical documentation; however, follow-up was not standardized due to the retrospective design. Long-term surveillance and recurrence assessment were limited to documented clinical encounters and were not predefined endpoints. |
| [6a. Eligibility criteria](https:/resources.equator-network.org/reporting-guidelines/strobe/items/eligibility-criteria.html?utm_source=strobe&utm_medium=checklist&utm_campaign=1_1) | **Cohort study:** Give the eligibility criteria, and the sources and methods of selection of participants. Describe methods of follow-up. **Case-control study:** Give the eligibility criteria, and the sources and methods of case ascertainment and control selection. Give the rationale for the choice of cases and controls. **Cross-sectional study:** Give the eligibility criteria, and the sources and methods of selection of participants. | **Location:**  - **Methods – “Patient Selection” (entire section)** - **Methods – “Study Design and Ethical Approval” (first paragraph)** - **Methods – “Data Collection” (follow-up description)**  **Statement:** The eligibility criteria are clearly defined in the “Patient Selection” section.  Inclusion criteria included:   - Age ≥ 18 years - Surgical resection for elastofibroma dorsi performed at the study institution - Histopathological confirmation of the diagnosis - Minimum one month of postoperative follow-up - Complete availability of imaging, operative, and pathology records   Exclusion criteria included:   - Diagnosis based solely on imaging or clinical findings without surgical confirmation - Inconclusive histopathology or alternative diagnosis - Missing essential data - Pediatric patients (<18 years) - Lack of documented informed consent   Participants were identified retrospectively through institutional surgical records and confirmed via pathology databases at a tertiary thoracic surgery department. Consecutive patients meeting eligibility criteria between January 2010 and June 2025 were included.  Follow-up data were obtained from routine outpatient clinical documentation and imaging records. Due to the retrospective design, follow-up was not standardized, and long-term surveillance was not protocol-driven. Recurrence assessment was limited to documented clinical encounters during routine care. |
| [6b. Matching criteria](https:/resources.equator-network.org/reporting-guidelines/strobe/items/matching-criteria.html?utm_source=strobe&utm_medium=checklist&utm_campaign=1_1) | **Cohort study:** For matched studies, give matching criteria and number of exposed and unexposed. **Case-control study:** For matched studies, give matching criteria and the number of controls per case. | **Location:**  - **Methods – Patient Selection** - (Implicitly confirmed throughout Methods section)  **Statement:** This was an unmatched retrospective cohort study. No matching procedures were applied. Participants were included consecutively based on predefined eligibility criteria, and no exposed/unexposed matching or case-control matching was performed. Therefore, this item is not applicable. |
| [7. Variables](https:/resources.equator-network.org/reporting-guidelines/strobe/items/variables.html?utm_source=strobe&utm_medium=checklist&utm_campaign=1_1) | Clearly define all outcomes, exposures, predictors, potential confounders, and effect modifiers. Give diagnostic criteria, if applicable. | **Location:**  - **Methods – “Variables and Groupings” (entire section)** - **Methods – “Postoperative Outcomes”** - **Methods – “Statistical Analysis”** - **Methods – “Patient Selection” (histopathological confirmation)**  **Statement:** All key variables are clearly defined in the Methods section.  **Primary outcomes:**   - Postoperative seroma formation - Length of hospital stay (LOS)   **Secondary outcomes:**   - Symptom burden (quantified using a composite “Symptom Count” score ranging from 0–4) - Bilateral disease status   **Exposures and predictors:**   - Age - Sex - Smoking status - Hand dominance - Occupational category and agricultural work - Tumor size parameters (pathological length, area, and volume) - Preoperative tumor measurements (radiological dimensions) - Symptom duration - Individual symptoms (pain, swelling, snapping, movement limitation)   **Potential confounders (included a priori in multivariable modeling):**   - Age - Sex - Smoking status   **Effect modifiers explored:**   - Bilaterality (in relation to symptom burden) - Hand dominance (in relation to lesion laterality)   **Diagnostic criteria:** All cases required histopathological confirmation of elastofibroma dorsi following surgical excision. Radiological findings were supportive but not used as sole diagnostic criteria.  Variables were defined using explicit operational definitions (e.g., Symptom Count scoring system; seroma defined as clinically detected postoperative fluid collection requiring observation or intervention; bilaterality defined as involvement of both scapulothoracic regions). |
| [8. Data sources / measurement](https:/resources.equator-network.org/reporting-guidelines/strobe/items/data-sources-measurement.html?utm_source=strobe&utm_medium=checklist&utm_campaign=1_1) | For each variable of interest give sources of data and details of methods of assessment (measurement). Describe comparability of assessment methods if there is more than one group. | **Location:**  - **Methods – “Data Collection”** - **Methods – “Variables and Groupings”** - **Methods – “Imaging Modalities”** - **Methods – “Surgical and Histopathologic Data”** - **Methods – “Postoperative Outcomes”**  **Statement:** The sources of data and methods of measurement for all variables are clearly described in the Methods section.  **Data sources:** Clinical and demographic data were extracted retrospectively from the institutional Health Information Management System (HIMS), surgical records, outpatient documentation, PACS imaging archives, and histopathology reports using a standardized data collection form. Two investigators independently extracted data, and discrepancies were resolved by consensus.  **Measurement of exposures and predictors:**   - **Age, sex, smoking status, occupational history, and hand dominance** were obtained from medical records. - **Symptoms (pain, swelling, snapping, movement limitation)** were determined based on documented clinical evaluation and patient-reported history. - **Symptom duration** was extracted from clinical documentation. - **Snapping phenomenon** was defined clinically as audible or palpable scapulothoracic friction during active shoulder abduction.   **Tumor size assessment:**   - **Preoperative tumor dimensions** were measured using CT and/or MRI images available in PACS, based on radiology reports. - **Pathological tumor dimensions** (length, width, depth) were obtained from postoperative histopathology reports. - Tumor surface area was calculated as the product of the longest and shortest axes. - Tumor volume was calculated as length × width × depth. - In bilateral cases, size-related parameters were summed to reflect total tumor burden per patient.   **Outcome assessment:**   - **Seroma** was identified based on postoperative clinical documentation and requirement for observation or intervention (e.g., aspiration). - **Length of hospital stay (LOS)** was calculated from the date of surgery to discharge.   **Comparability of measurement methods:** All patients underwent histopathological confirmation, ensuring uniform diagnostic criteria across the cohort. Radiological measurements were derived from routine clinical imaging and may reflect variability inherent to imaging modality (CT vs MRI) and retrospective documentation. Pathological measurements were considered the reference standard for tumor size analysis. All analyses were conducted at the patient level using consistent operational definitions across groups (symptomatic vs asymptomatic; unilateral vs bilateral). |
| [9. Bias](https:/resources.equator-network.org/reporting-guidelines/strobe/items/bias.html?utm_source=strobe&utm_medium=checklist&utm_campaign=1_1) | Describe any efforts to address potential sources of bias. | **Location:**  - **Methods – “Study Design and Ethical Approval” (limitations of retrospective design)** - **Methods – “Data Collection” (dual data extraction)** - **Methods – “Patient Selection” (consecutive inclusion)** - **Discussion – Limitations section (selection bias, measurement bias, confounding)**  **Statement:** Potential sources of bias were acknowledged and partially addressed in both the Methods and Discussion sections.  **Selection bias:** To minimize selection bias, all consecutive adult patients who underwent surgical resection for histopathologically confirmed elastofibroma dorsi between January 2010 and June 2025 were included. However, as a surgically treated cohort, the study inherently reflects a selected population and does not represent conservatively managed cases.  **Information and measurement bias:** Data were extracted using a standardized collection form from institutional electronic systems. Two independent investigators performed data extraction, and discrepancies were resolved by consensus to improve data reliability. Histopathological confirmation was required for all cases, ensuring diagnostic uniformity. Pathological measurements were used as reference standards for tumor size to reduce imaging-related variability.  **Confounding:** Multivariable logistic regression analysis was performed to adjust for clinically relevant covariates (age, sex, and smoking status) when evaluating predictors of bilateral disease. Pre-specification of covariates aimed to reduce residual confounding.  **Retrospective design bias:** The retrospective nature of the study may introduce record-based bias and limits control over unmeasured variables (e.g., BMI, detailed workload quantification, medication use). This limitation is explicitly acknowledged in the Discussion.  **Follow-up bias:** Follow-up was based on routine clinical documentation and was not standardized, which may limit long-term outcome assessment. Recurrence was therefore not used as a primary endpoint. |
| [10. Study size](https:/resources.equator-network.org/reporting-guidelines/strobe/items/study-size.html?utm_source=strobe&utm_medium=checklist&utm_campaign=1_1) | Explain how the study size was arrived at. | **Location:**  - **Methods – “Patient Selection” (first paragraph)** - **Results – first paragraph (cohort description)** - **Discussion – Limitations (implicit clarification of design)**  **Statement:** The study size was determined by the total number of consecutive adult patients who underwent surgical resection for histopathologically confirmed elastofibroma dorsi at the study institution between January 2010 and June 2025. No formal sample size or power calculation was performed due to the retrospective cohort design. The final sample consisted of all eligible cases meeting predefined inclusion and exclusion criteria during the 15-year study period (N = 102). |
| [11. Quantitative variables](https:/resources.equator-network.org/reporting-guidelines/strobe/items/quantitative-variables.html?utm_source=strobe&utm_medium=checklist&utm_campaign=1_1) | Explain how quantitative variables were handled in the analyses. If applicable, describe which groupings were chosen, and why. | **Location:**  - **Methods – “Variables and Groupings”** - **Methods – “Statistical Analysis”** - **Results – Symptom Burden section**  **Statement:** Quantitative variables were handled according to their distribution characteristics. Normality was assessed using the Shapiro–Wilk test.   - Normally distributed variables were presented as mean ± standard deviation and analyzed using parametric tests. - Non-normally distributed variables were summarized as median (interquartile range) and analyzed using non-parametric tests.   Continuous variables (e.g., age, tumor dimensions, symptom duration, length of hospital stay) were analyzed both as continuous measures and, where clinically relevant, within predefined comparison groups (e.g., unilateral vs bilateral; symptomatic vs asymptomatic).  Tumor size parameters (length, area, volume) were analyzed as continuous variables. In bilateral cases, values were aggregated to represent total tumor burden at the patient level.  A composite “Symptom Count” variable (range 0–4) was constructed to quantify symptom burden. This variable was analyzed both as a continuous ordinal measure (Spearman correlation) and categorically to compare burden across clinical subgroups.  Age was analyzed as a continuous variable in regression modeling to preserve statistical power and avoid arbitrary categorization. |
| [12a. Statistical methods](https:/resources.equator-network.org/reporting-guidelines/strobe/items/statistical-methods-description.html?utm_source=strobe&utm_medium=checklist&utm_campaign=1_1) | Describe all statistical methods, including those used to control for confounding. | **Location:**  - Methods – “Statistical Analysis” - Results – “Independent Predictors of Bilateral Disease”  **Statement:** All statistical analyses were performed using R software (version 4.5.1). Normality of continuous variables was assessed using the Shapiro–Wilk test. Continuous variables were summarized as mean ± standard deviation or median (interquartile range), as appropriate. Group comparisons were conducted using the Independent Samples t-test or Mann–Whitney U test for two-group comparisons, and one-way ANOVA or Kruskal–Wallis test for multiple groups.  Associations between continuous or ordinal variables were evaluated using Spearman’s rank correlation coefficient.  To control for potential confounding, multivariable logistic regression analysis was performed. Bilateral disease was defined as the dependent variable. Age, sex, and smoking status were included a priori as clinically relevant covariates. Adjusted odds ratios (ORs) with 95% confidence intervals (CIs) were reported.  All analyses were conducted at the patient level. A p-value < 0.05 was considered statistically significant. Effect estimates and confidence intervals were reported to emphasize the magnitude of associations beyond p-values alone. |
| [12b. Statistical methods – subgroups and interactions](https:/resources.equator-network.org/reporting-guidelines/strobe/items/statistical-methods-subgroups-interactions.html?utm_source=strobe&utm_medium=checklist&utm_campaign=1_1) | Describe any methods used to examine subgroups and interactions. | **Location:**  - Results – Symptomatic vs Asymptomatic - Results – Unilateral vs Bilateral - Results – Occupational groups - Results – Symptom-specific analyses  **Statement:** Predefined subgroup analyses were conducted to compare symptomatic versus asymptomatic patients and unilateral versus bilateral cases. Additional subgroup analyses were performed according to occupational category, hand dominance, and individual symptom presence (pain, swelling, snapping, movement limitation).  No formal interaction terms were included in the multivariable regression model. Subgroup comparisons were exploratory and hypothesis-generating. |
| [12c. Statistical methods – missing data](https:/resources.equator-network.org/reporting-guidelines/strobe/items/statistical-methods-missing-data.html?utm_source=strobe&utm_medium=checklist&utm_campaign=1_1) | Explain how missing data were addressed. | **Location:**  - Methods – Patient Selection - Data Collection section  **Statement:** Cases with missing essential clinical, imaging, pathological, or follow-up data were excluded based on predefined exclusion criteria. Only patients with complete baseline and early postoperative data were included in the final analysis. As a result, no imputation techniques were required, and complete-case analysis was performed. |
| [12di. Statistical methods – loss to follow-up](https:/resources.equator-network.org/reporting-guidelines/strobe/items/statistical-methods-loss-to-follow-up.html?utm_source=strobe&utm_medium=checklist&utm_campaign=1_1) | **Cohort study:** If applicable, describe how loss to follow-up was addressed. | **Location:**  - Methods – Patient Selection - Postoperative Outcomes  **Statement:** Because the primary endpoints were early postoperative outcomes (seroma formation and length of hospital stay), all included patients had complete in-hospital follow-up. No patients were lost to follow-up for early postoperative outcomes.  Long-term follow-up was not standardized due to the retrospective design; therefore, recurrence and long-term outcomes were not analyzed as formal study endpoints. |
| [12dii. Statistical methods – matching cases and controls](https:/resources.equator-network.org/reporting-guidelines/strobe/items/statistical-methods-matching-cases-controls.html?utm_source=strobe&utm_medium=checklist&utm_campaign=1_1) | **Case-control study:** If applicable, explain how matching of cases and controls was addressed. | Not applicable. This was not a matched case-control study. |
| [12diii. Statistical methods – sampling strategy](https:/resources.equator-network.org/reporting-guidelines/strobe/items/statistical-methods-analytical-methods-sampling-strategy.html?utm_source=strobe&utm_medium=checklist&utm_campaign=1_1) | **Cross-sectional study:** If applicable, describe analytical methods taking account of sampling strategy. | Not applicable. This was not a cross-sectional study with sampling strategy. |
| [12e. Statistical methods – sensitivity analyses](https:/resources.equator-network.org/reporting-guidelines/strobe/items/statistical-methods-sensitivity-analyses.html?utm_source=strobe&utm_medium=checklist&utm_campaign=1_1) | Describe any sensitivity analyses. | No formal sensitivity analyses were performed. All multivariable models were prespecified using clinically relevant covariates (age, sex, smoking status) selected a priori. Findings should therefore be interpreted within the constraints of the retrospective design. |
| **Results** |  |  |
| [13a. Participant numbers](https:/resources.equator-network.org/reporting-guidelines/strobe/items/participants-numbers.html?utm_source=strobe&utm_medium=checklist&utm_campaign=1_1) | Report the numbers of individuals at each stage of the study—e.g., numbers potentially eligible, examined for eligibility, confirmed eligible, included in the study, completing follow-up, and analysed; Consider use of a flow diagram. | **Location:**  - Methods – “Patient Selection” - Results – First paragraph  **Statement:** All consecutive adult patients who underwent surgical resection for histopathologically confirmed elastofibroma dorsi at our institution between January 2010 and June 2025 were screened for eligibility.  A total of 102 patients met the predefined inclusion criteria and were included in the final analysis. All included patients had complete baseline, surgical, pathological, and early postoperative data.  All 102 patients completed in-hospital follow-up for the primary postoperative endpoints (seroma formation and length of hospital stay) and were included in the final statistical analysis. |
| [13b. Participants – non-participation](https:/resources.equator-network.org/reporting-guidelines/strobe/items/participants-non-participation.html?utm_source=strobe&utm_medium=checklist&utm_campaign=1_1) | Give reasons for non-participation at each stage. | **Location:**  - Methods – “Patient Selection”  **Statement:** During the study period, patients who did not meet inclusion criteria were excluded due to one or more of the following reasons: absence of surgical resection, lack of histopathological confirmation, missing essential clinical or imaging data, pediatric age (<18 years), or lack of documented informed consent.  No eligible surgically treated patients meeting the inclusion criteria were excluded from the final analysis. |
| [13c. Participants – flow diagram](https:/resources.equator-network.org/reporting-guidelines/strobe/items/participants-flow-diagram.html?utm_source=strobe&utm_medium=checklist&utm_campaign=1_1) | Consider use of a flow diagram. | Although a formal flow diagram was not included, the participant selection process is explicitly described in the Methods section. All consecutive eligible surgically treated patients during the study period were included in the final analysis without loss to follow-up for early postoperative outcomes. |
| [14a. Descriptive data – participant characteristics](https:/resources.equator-network.org/reporting-guidelines/strobe/items/descriptive-data-participant-characteristics.html?utm_source=strobe&utm_medium=checklist&utm_campaign=1_1) | Give characteristics of study participants (e.g., demographic, clinical, social) and information on exposures and potential confounders. Present the information in a table. | **Location:**  - Results – First paragraph - Table 1 - Table 2 - Table 3 - Table 4  **Statement:** Baseline demographic, clinical, occupational, and pathological characteristics of the study population are presented in **Table 1**. These include age, sex, smoking status, comorbidities, occupational categories, symptom profiles, tumor size parameters, and bilaterality status.  Comparative descriptive data are further presented in:   - **Table 2:** Symptomatic vs asymptomatic patients - **Table 3:** Unilateral vs bilateral cases - **Table 4:** Occupational groups   Potential confounders (age, sex, smoking status) and primary exposures (tumor size parameters, bilaterality, symptom duration, occupational status) are clearly reported. |
| [14b. Descriptive data – missing data](https:/resources.equator-network.org/reporting-guidelines/strobe/items/descriptive-data-missing-data.html?utm_source=strobe&utm_medium=checklist&utm_campaign=1_1) | Indicate the number of participants with missing data for each variable of interest. | **Location:**  - Methods – Patient Selection - Data Collection  **Statement:** Only patients with complete essential clinical, imaging, surgical, and pathological data were included in the final analysis. Cases with missing essential data were excluded based on predefined criteria.  As a result, no missing data were present for the primary outcomes or key covariates included in the analyses, and complete-case analysis was performed. |
| [14c. Descriptive data – follow-up time](https:/resources.equator-network.org/reporting-guidelines/strobe/items/descriptive-data-follow-up-time.html?utm_source=strobe&utm_medium=checklist&utm_campaign=1_1) | **Cohort study:** Summarise follow-up time—e.g., average and total amount. | **Location:**  - Methods – Postoperative Outcomes - Results – LOS section  **Statement:** Follow-up for primary endpoints was limited to the in-hospital postoperative period. All included patients completed early postoperative follow-up until discharge.  The mean length of hospital stay was **5.4 ± 3.5 days**.  Long-term follow-up was not standardized due to the retrospective design, and recurrence was not evaluated as a primary endpoint. |
| [15. Outcome data](https:/resources.equator-network.org/reporting-guidelines/strobe/items/outcome-data.html?utm_source=strobe&utm_medium=checklist&utm_campaign=1_1) | **Cohort study:** Report numbers of outcome events or summary measures over time. **Case-control study:** Report numbers in each exposure category, or summary measures of exposure. **Cross-sectional study:** Report numbers of outcome events or summary measures. | **Location:**  - Results – Postoperative Outcomes - Table 6 - Table 7  **Statement:** The overall incidence of postoperative seroma was **18.6% (n = 19)**.  Length of hospital stay had a mean of **5.4 ± 3.5 days**.  Comparative analyses of seroma development are presented in **Table 6**, and factors associated with length of hospital stay are presented in **Table 7**.  Outcome data are reported both as absolute event numbers and as summary measures with corresponding statistical tests. |
| [16a. Main results](https:/resources.equator-network.org/reporting-guidelines/strobe/items/main-results.html?utm_source=strobe&utm_medium=checklist&utm_campaign=1_1) | Give unadjusted estimates and, if applicable, confounder-adjusted estimates and their precision (e.g., 95% confidence intervals). Make clear which confounders were adjusted for and why they were included. | **Location:**  - Results – Independent Predictors of Bilateral Disease  **Statement:** Unadjusted comparisons were performed using appropriate parametric and non-parametric tests.  To control for confounding, multivariable logistic regression analysis was performed with bilateral disease as the dependent variable.  The model included:   - Age - Sex - Smoking status   Female sex was independently associated with bilateral disease (male sex OR = 0.136; 95% CI 0.033–0.564; p = 0.008).  Younger age was also independently associated with bilaterality (OR = 0.933 per year; 95% CI 0.881–0.988; p = 0.017).  Smoking status was not significantly associated (p = 0.355).  Both unadjusted and adjusted estimates are clearly reported with 95% confidence intervals. |
| [16b. Main results – category boundaries](https:/resources.equator-network.org/reporting-guidelines/strobe/items/main-results-category-boundaries.html?utm_source=strobe&utm_medium=checklist&utm_campaign=1_1) | Report category boundaries when continuous variables were categorised. | Continuous variables (e.g., age, tumor size, symptom duration) were primarily analyzed as continuous measures to preserve statistical power and avoid arbitrary categorization.  Categorical groupings used in subgroup analyses (e.g., symptomatic vs asymptomatic; unilateral vs bilateral; occupational categories) were predefined based on clinical criteria rather than statistical cutoffs.  No arbitrary statistical dichotomization of continuous variables was performed. |
| [16c. Main results – risk](https:/resources.equator-network.org/reporting-guidelines/strobe/items/main-results-risk.html?utm_source=strobe&utm_medium=checklist&utm_campaign=1_1) | If relevant, consider translating estimates of relative risk into absolute risk for a meaningful time period. | Absolute event rates were reported for postoperative seroma (18.6%) and bilateral disease (50.0%).  Given the observational design and lack of time-to-event analysis, translation of relative risk into time-based absolute risk was not applicable. |
| [17. Other analyses](https:/resources.equator-network.org/reporting-guidelines/strobe/items/other-analyses.html?utm_source=strobe&utm_medium=checklist&utm_campaign=1_1) | Report other analyses done—e.g., analyses of subgroups and interactions, and sensitivity analyses. | **Location:**  - Results – Symptomatic vs Asymptomatic - Results – Occupational Groups - Results – Symptom Burden - Methods – Statistical Analysis  **Statement:** Predefined subgroup analyses were conducted comparing:   - Symptomatic vs asymptomatic patients - Unilateral vs bilateral disease - Occupational categories - Individual symptom profiles   Spearman correlation analyses were used to evaluate associations between symptom burden and tumor dimensions.  No formal interaction terms were included in regression modeling. No sensitivity analyses were performed.  Subgroup analyses were exploratory and hypothesis-generating. |
| **Discussion** |  |  |
| [18. Key results](https:/resources.equator-network.org/reporting-guidelines/strobe/items/key-results.html?utm_source=strobe&utm_medium=checklist&utm_campaign=1_1) | Summarise key results with reference to study objectives. | **Location:**  - Discussion – First 2 paragraphs - Conclusion  **Statement:** The key findings are summarized at the beginning of the Discussion and aligned with the predefined study objectives. The study demonstrated that:   1. Greater disease burden—particularly bilaterality and larger pathological tumor dimensions—was associated with increased symptom burden. 2. Female sex and younger age were independently associated with bilateral disease. 3. Lesion laterality strongly correlated with hand dominance, supporting a potential mechanical contribution. 4. Postoperative seroma formation was associated with symptomatic presentation and longer symptom duration rather than tumor size. 5. Length of hospital stay correlated primarily with tumor size.   These findings directly address the prespecified objectives and hypotheses stated in the Background section. |
| [19. Limitations](https:/resources.equator-network.org/reporting-guidelines/strobe/items/limitations.html?utm_source=strobe&utm_medium=checklist&utm_campaign=1_1) | Discuss limitations of the study, taking into account sources of potential bias or imprecision. Discuss both direction and magnitude of any potential bias. | **Location:**  - Discussion – Limitations section (final paragraphs)  **Statement:** Several limitations are acknowledged:   1. **Selection bias:** The study includes only surgically treated patients. This may overrepresent symptomatic or larger lesions, potentially inflating associations between tumor size and symptom burden. The magnitude of this bias may limit extrapolation to conservatively managed ED cases. 2. **Retrospective design:** Record-based data collection introduces potential information bias and restricts control over unmeasured confounders (e.g., BMI, workload intensity, medication use). This may attenuate or exaggerate observed associations. 3. **Single-center setting:** Regional occupational patterns (e.g., homemakers, agricultural workers) may influence lesion distribution and morphology, limiting generalisability. 4. **Measurement variability:** Radiological measurements were derived from routine imaging reports rather than standardized 3D segmentation. Minor underestimation on imaging compared to pathology may influence effect size estimation. 5. **No long-term outcomes:** Recurrence and functional outcomes were not systematically assessed, limiting conclusions regarding long-term benefit. 6. **No sensitivity analyses or interaction modeling:** This may limit robustness testing of multivariable findings.   The direction of bias likely favors stronger associations between disease burden and symptoms due to surgical cohort enrichment. |
| [20. Interpretation](https:/resources.equator-network.org/reporting-guidelines/strobe/items/interpretation.html?utm_source=strobe&utm_medium=checklist&utm_campaign=1_1) | Give a cautious overall interpretation considering objectives, limitations, multiplicity of analyses, results from similar studies, and other relevant evidence. | **Location:**  - Discussion – Entire section - Conclusion  **Statement:** The findings are interpreted cautiously in the context of the study objectives and limitations.  The observed association between bilaterality, pathological size, and symptom burden supports the hypothesis that mechanical and structural disease burden contributes to clinical manifestation. The strong correlation between lesion laterality and hand dominance further reinforces the plausibility of repetitive microtrauma.  However, given the retrospective design and multiple subgroup analyses, these findings should be considered hypothesis-generating rather than definitive evidence of causality.  The results are consistent with previously published case series reporting female predominance, bilateral variability, and size-related symptom correlation, while adding multivariable-adjusted evidence and pathology-based measurements.  Interpretation avoids overstatement and does not claim therapeutic superiority or causal inference. |
| [21. Generalisability](https:/resources.equator-network.org/reporting-guidelines/strobe/items/generalisability.html?utm_source=strobe&utm_medium=checklist&utm_campaign=1_1) | Discuss the generalisability (external validity) of the study results. | **Location:**  - Discussion – Final limitation paragraphs  **Statement:** The study reflects a surgically treated cohort from a tertiary referral center and may not represent the full clinical spectrum of radiologically diagnosed, conservatively managed elastofibroma dorsi.  Regional occupational distribution and referral patterns may limit applicability to populations with different mechanical exposure profiles.  Nevertheless, the large single-center cohort, standardized histopathological confirmation, and consistent patient-level analysis enhance internal validity and provide clinically relevant insights for centers managing surgically treated ED. |
| **Other information** |  |  |
| [22. Funding](https:/resources.equator-network.org/reporting-guidelines/strobe/items/funding.html?utm_source=strobe&utm_medium=checklist&utm_campaign=1_1) | Give the source of funding and the role of the funders for the present study and, if applicable, for the original study on which the present article is based. | **Location:**  - Declarations – Funding  **Statement:** The authors received no specific funding for this study. No external funding sources were involved in study design, data collection, analysis, interpretation, manuscript preparation, or decision to submit the manuscript. |

## 1 How to specify where content is

Tell the reader where they can find information. E.g.,

- Results; paragraph 2
- Methods, Participants; paragraphs 1 & 2.
- Table 3
- Supplement B, para. 4

If you have chosen not to describe an item, explain why. You can do this in the checklist, or as a note below it.

You can describe items in the article body, or in tables, figures, or supplementary materials, and should prioritize items you feel are most important to your intended audience. The order of items in your manuscript does not need to match the order of items in this checklist. You can decide how best to structure your work.

## 2 How to cite

Describe how you used STROBE at the end of your Methods section, referencing the resources you used e.g.,

‘We used the STROBE reporting guideline(1) to draft this manuscript, and the STROBE reporting checklist(2) when editing, included in supplement A’

If you use a reporting checklist, remember to include it as a supplement when publishing so that readers can easily find information and see how you have interpreted the guidance.

1. Elm E von, Altman DG, Egger M, Pocock SJ, Gøtzsche PC, Vandenbroucke JP, et al. The strengthening the reporting of observational studies in epidemiology (STROBE) statement: Guidelines for reporting observational studies. Annals of Internal Medicine [Internet]. 2007 Oct;147(8):573–7. Available from: <https://www.acpjournals.org/doi/10.7326/0003-4819-147-8-200710160-00010>

2. Elm E von, Altman DG, Egger M, Pocock SJ, Gøtzsche PC, Vandenbroucke JP, et al. The STROBE reporting checklist. In: Harwood J, Albury C, Beyer J de, Schlüssel M, Collins G, editors. The EQUATOR network reporting guideline platform [Internet]. The UK EQUATOR Centre; 2025. Available from: [https:/resources.equator-network.org/reporting-guidelines/strobe/strobe-checklist.docx](https://https:/resources.equator-network.org/reporting-guidelines/strobe/strobe-checklist.docx)
